# Vagus Nerve Stimulation and Its Cardioprotective Abilities: A Systematic Review

**DOI:** 10.3390/jcm12051717

**Published:** 2023-02-21

**Authors:** Ahmed Banibella Abdelmagied Elamin, Kowthar Forsat, Solomon Silas Senok, Nandu Goswami

**Affiliations:** 1 College of Medicine, Ajman University, Ajman P.O. Box 346, United Arab Emirates; 2 Institute of Physiology (Gravitational Physiology and Medicine), Medical University of Graz, 8036 Graz, Austria; 3 College of Medicine, Mohammed Bin Rashid University of Medicine and Health Sciences, Dubai P.O. Box 505055, United Arab Emirates

**Keywords:** atrial fibrillation, autonomic nervous system, cardiac arrest, heart failure, ischemia/reperfusion injury, selective vagus nerve stimulation, vagus nerve stimulation, ventricular fibrillation

## Abstract

Despite the vagus nerve stimulator (VNS) being used in neuroscience, it has recently been highlighted that it has cardioprotective functions. However, many studies related to VNS are not mechanistic in nature. This systematic review aims to focus on the role of VNS in cardioprotective therapy, selective vagus nerve stimulators (sVNS), and their functional capabilities. A systemic review of the current literature was conducted on VNS, sVNS, and their ability to induce positive effects on arrhythmias, cardiac arrest, myocardial ischemia/reperfusion injury, and heart failure. Both experimental and clinical studies were reviewed and assessed separately. Of 522 research articles retrieved from literature archives, 35 met the inclusion criteria and were included in the review. Literature analysis proves that combining fiber-type selectivity with spatially-targeted vagus nerve stimulation is feasible. The role of VNS as a tool for modulating heart dynamics, inflammatory response, and structural cellular components was prominently seen across the literature. The application of transcutaneous VNS, as opposed to implanted electrodes, provides the best clinical outcome with minimal side effects. VNS presents a method for future cardiovascular treatment that can modulate human cardiac physiology. However, continued research is needed for further insight.

## 1. Introduction

The vagus nerve (VN) is the most diverse cranial nerve with extensive distribution in the body, originating in the medulla [1]. It has many clinical functions as the main parasympathetic component of the human body, controlling mood, digestion, the immune response, and the heart [2]. The effects of the vagus on the heart can be predicted based on its anatomical distribution, and they include negative chronotropic (reduction in heart rate), dromotropic (reduction in atrioventricular conduction), and inotropic (reduction in ventricular contractility) actions [3]. The human vagal nerve is similar to that of the pig in terms of its diameter, multiple fasciae, and fibrous tissue structures. Unsurprisingly, the evidence generated from these pig studies suggests that vagal nerve modulations could play an essential role in future clinical trials [4]. Function-specific (organotopic) organizations in the cervical vagus nerve have strengthened the possibility of selective electrical neuromodulation with minimal side effects and enhanced organ selectivity [5]. These effects are widely applied to heart diseases such as heart failure [6]. Indeed, VNS has been used as a possible therapeutic tool for other heart conditions such as myocardial infarction (MI), atrial fibrillation (AF), and cardiac arrest [3,7,8].

American neurologist James Corning developed the first VNS in the late 19th century to treat epilepsy [3]. Later in 1997, the first VNS device was approved by the US Food and Drug Administration (FDA) for the treatment of resistant partial-onset seizures [9,10]. The term VNS refers to any form of stimulation of the vagus nerve, whether through invasive or non-invasive techniques [11]. More risks and side effects accompany the surgical method of stimulating the vagus nerve; therefore, the transcutaneous stimulation process is more desired [12,13]. The latest devices used for therapeutic VNS are AspireSR^®^ and SenTiva™, as they are currently being implemented in treating epilepsy [14]. The most used models in research and experiments are gammaCore, electroCore, or NemosCerbomed [12,15,16,17,18,19]. Some practical uses for VNS have been noticed in sepsis, pain management, obesity, diabetes, cardiovascular diseases, ventilator-induced lung injury (VILI), other lung injuries, stroke, traumatic brain injury, and rheumatoid arthritis [9,10]. Although VNS is safe and a widely tolerable therapeutic approach, some minor side effects have been observed [12]. These side effects were reported in several studies as pain and tingling at the site of stimulation [20,21]. However, they are usually stimulation-related and tend to be reversible with time [22]. Less common side effects reported in less than 1% of the population are headache, dizziness, nausea, vomiting, facial drooping, vocal hoarseness, heart palpitations, and nasopharyngitis [12]. To increase the selective actions of VNS, many selective VNS (sVNS) models have been developed.

### 1.1. sVNS Models

The models for VNS have varying effects depending on the specific nerve fibers they target [10]. In addition to being more clinically effective, they have fewer side effects when compared to non-selective vagus nerve stimulation (nsVNS) [23]. A 2018 study done by Dali et al. presented a 62% reduction in side effects for cardiac modulation in sheep using sVNS compared to nsVNS [24]. However, the author did not specify these side effects, and the effectiveness of the sVNS configuration in comparison to nsVNS was unclear.

#### 1.1.1. Fiber Selective Stimulation

Fiber-selective stimulation manipulates different vagus nerve fiber thresholds to activate the desired (selected) fibers [10]. This is achieved by the implantation of cuff arrays around the cervical vagus nerve surgically under general anesthesia [22]. It presents three different methods, which include: anodal block, depolarizing pre-pulses, and slowly rising pulses [25].

#### 1.1.2. Spatially Selective Stimulation

Spatially-selective VNS aims at stimulating a specific area of the nerve cross-section, activating particular (selected) fascicles [10]. It is the preferred technique when activating larger nerve fibers that are difficult to avoid [23].

#### 1.1.3. Kiliohertz Electrical Stimulation (KES)

KES block is a technique in which the propagation of action potentials in nerves is inhibited by electrical stimulations of a value not less than 5 kilohertz (kHz) [10], affecting the entire nerve and providing directional innervation.

#### 1.1.4. Neural Titration

Neural titration acts by activating both the afferent and efferent fibers of the vagus nerve, whilst having a higher affinity for efferent fibers which results in its desired affects [26].

#### 1.1.5. Transcutaneous—Vagus Nerve Stimulation (tVNS)

**Figure 1:**
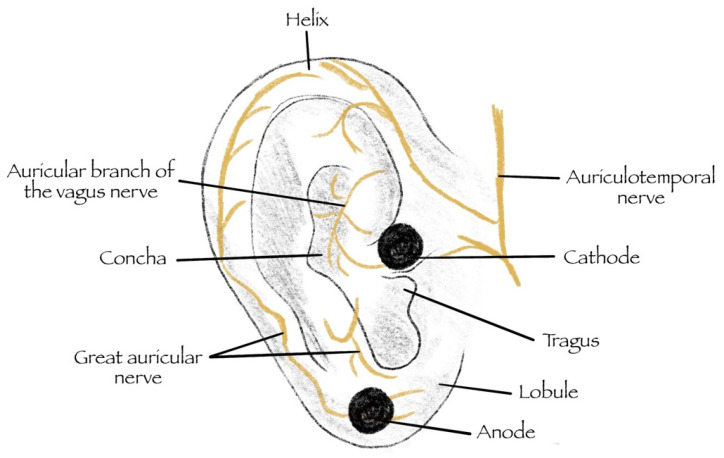
LLTS involves the use of an anode and a cathode to deliver tragus stimulation to the afferent fibers of the vagus nerve (auricular branch). The stimulation charge then enters the medulla oblongata at the brain stem, in turn exciting the efferent fibers of the vagus nerve and enabling the desired cardiac neuromodulation [27].

Low Level Tragus Stimulation (LLTS) provides the neural innervation desired from the auricular branch of the vagus nerve (seen in Figure 1), towards the medulla oblongata at the brainstem (reflex center), and finally, the efferent vagal fibers arriving at the heart [27]. Alternatively, tVNS of the cymbal concha provides a different route for VNS. This model provides the most potential, with desired attenuation of arrhythmogenic and heart failure effects on the myocardium. It also acts as the most clinically viable form of sVNS.

**More information on the models is presented in the results.**

This review systematically examines the current clinical applicability of sVNS in cardioprotective function, focusing on the mechanisms involved and discussing future clinical applications. It also focuses on recently developed models based on recent advancements in vagal nerve physiology and interventions, with the hopes of using them against cardiovascular disease.

## 2. Materials and Methods

A systematic review was performed on the effect of VNS application for cardiac therapy following the Preferred Reporting Items for Systematic Reviews and Meta-Analyses (PRISMA) guidelines [28]. The literature search did not include papers published before 2000 except two vital papers from 1983. This is due to recent improvements in neuromodulation and its application and usage of VNS. There was a restriction to cardiological-based pathologies and articles written in English. Studies analyzed include human observational studies and animal models. Papers were excluded due to being review articles, duplications, and lacking proper scientific basis or reliability of data collection. Data extraction was performed using the major electronic databases, including PubMed, Google Scholar, and Scopus.

**Figure 2:**
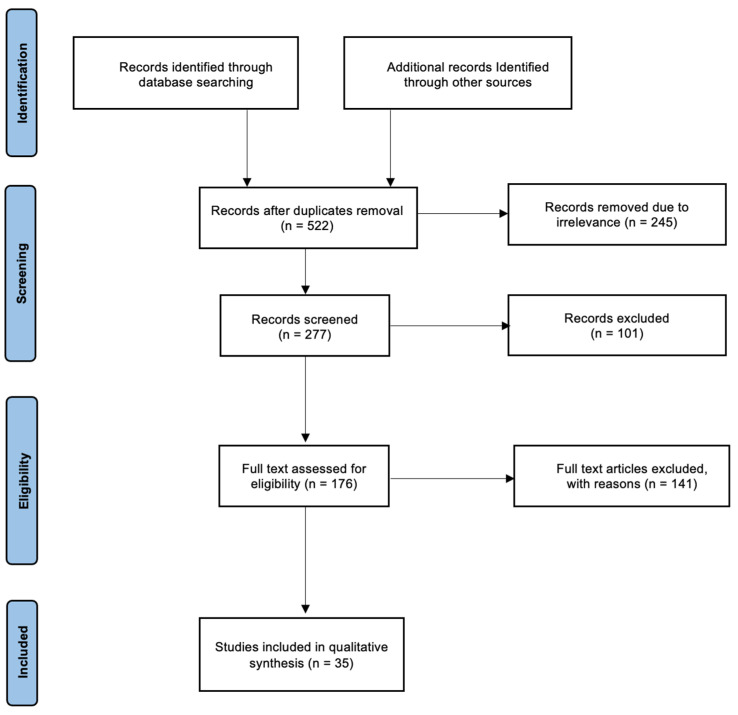
PRISMA flow diagram.

Moreover, other credible literature sources were also analyzed. Structured literature searches were conducted based on specific keywords, abbreviations, and inclusion criteria. MeSH keywords and search terms that were used include: “atrial fibrillation”, “autonomic nervous system”, “cardiac arrest”, “heart failure”, “myocardial ischemia”, “selective vagus nerve stimulation”, “vagus nerve stimulation”, and “ventricular fibrillation”. Boolean operators–AND, OR, NOT–were implemented. At first, the title and abstract were assessed to confirm that the topics match. After that, the articles were evaluated to prove the relevance of the articles to the current study. Data extracted from each paper eligible for the criteria were used for this study. The variables include study author and publication year, study setting, sample characteristics, studies exploring the effect of VNS on cardiac pathologies, and studies investigating effects of different parameters to deliver VNS. The data collection process is summarized in a PRISMA flowchart presented in Figure 2.

## 3. Results

The number of studies collected in the initial search after duplicate removal was 522 papers. A total of 245 papers were removed due to their irrelevance to this review. After screening the remaining papers according to the inclusion and exclusion criteria and considering both human and animal studies, a total of 35 papers were eligible and included in this review. The included research articles were categorized accordingly and are discussed further below in individual sections as follows:

Studies on VNS usage for myocardial infarction (n = 6 articles included and summarized in Table 1). Studies on VNS usage for atrial fibrillation, ventricular fibrillation, and heart rate modulation (n = 8 articles included and summarized in Table 2). Studies on VNS usage for cardiac arrest (n = 3 articles included and summarized in Table 3). Studies on VNS usage for heart failure (n = 9 articles included and summarized in Table 4). Studies on different sVNS techniques in various applications (n = 9 articles included and summarized in Table 5).

**Figure 3:**
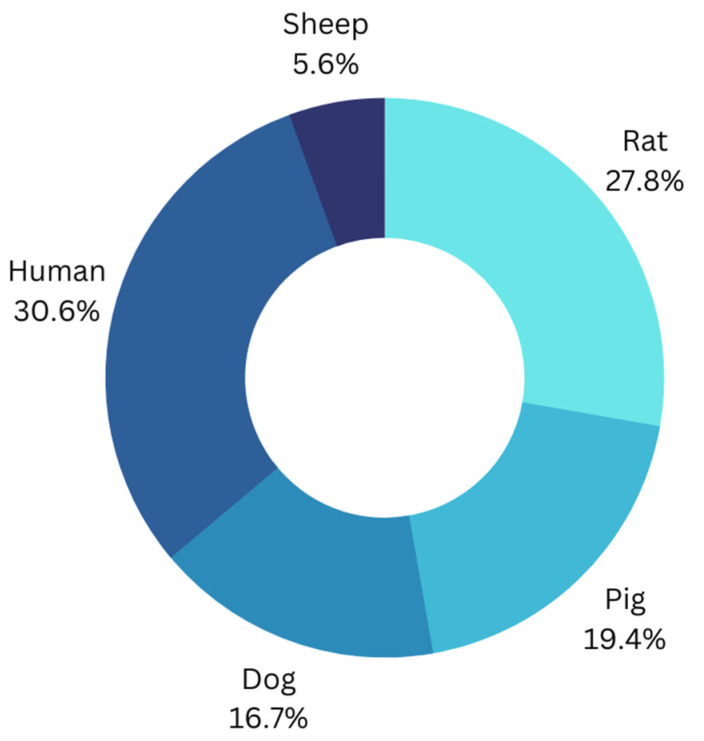
This pie chart represents the percentages of the different models used among the studies (n = 35).

**Figure 4:**
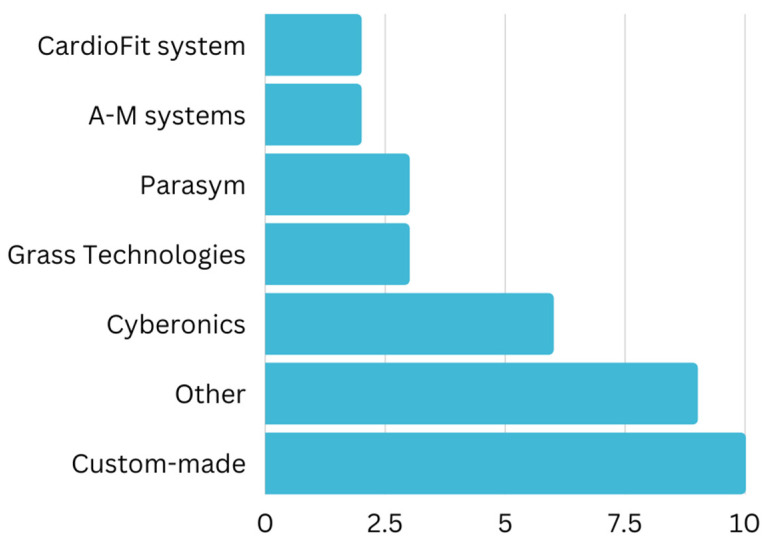
This bar chart represents the frequency of various VNS devices used among the studies (n = 35).

These 35 studies include experiments of VNS on humans (n = 11 studies), rats (n = 10 studies), pigs (n = 7 studies), dogs (n = 6 studies), and sheep (n = 2 studies) shown in Figure 3. The devices used for achieving VNS were mostly made by Cyberonics Inc. (Figure 4). Most of the studies used custom-made generators for the purpose of their experiments. The most common frequency used for achieving VNS was 20 Hz in 11 studies and 10 Hz in 6 studies. Pulse width used in these studies ranged between 0.2–10 ms, with the average being 0.7 ms. Pulse duration ranged between 50–500 μs, with the average being around 200 μs. However, most of the studies used 500 μs as the pulse width.

### 3.1. VNS Trial in Cardiac Diseases

#### 3.1.1. Myocardial Ischemia

A research experiment involved rats subjected to ischemia for 30 min followed by 4 h of reperfusion [29]. Results after the reperfusion showed that VNS inhibited the increased secretions elicited by I/R injury, which include products like Tumor necrosis factor-alpha (TNF-α), Interleukin-6 (IL-6), High mobility group box-1 (HMGB-1) and Interleukin-17A (IL-17A) [29,60]. Further findings demonstrated that activation of the cholinergic anti-inflammatory pathway (CAP) by the efferent vagal nerve causes the release of ACh that chains with the α7 nicotinic ACh receptors (α7nAChR) of macrophages, which inhibits the secretions of pro-inflammatory products like TNF-α [61]. In an experiment on rats with induced AMI, it was discovered that VNS weakened the AMI-induced increase in TNF-α expression [34]. VNS inhibited mitochondrial dysfunction by activating the phosphatidylinositol 3-kinase/protein kinase B (PI3K/Akt) signaling pathway, which prevents apoptosis of cardiomyocytes from the I/R injury [62]. These findings in rodents suggest that VNS application attenuates mitochondrial and ROS components of myocardial injury. In addition, VNS application in canines reduced serum TNF-α and IL-6 concentrations [31]. Moreover, increased expressions of ACh and α7nAChR in myocardial tissues after 2-h ischemia/reperfusion were seen [31]. More evidence has shown attenuated mitochondrial ROS production by VNS in pigs, with significantly reduced ventricular fibrillation and infarct size (at around 59%) [32]. Moreover, a study was performed on guinea pigs who underwent VNS implantation on the right cervical vagus nerve with induced myocardial infarction. Results demonstrated focal mitigation of remodeling for the intrinsic cardiac nervous system, with preserved myocytes (enhanced glycogenolysis) and maintained proapoptotic Bcl-2-associated X proteins [30]. The many modes of action presented by VNS warrant the potential for clinical trials for this modality to protect the myocardium that is at risk of I/R injury.

#### 3.1.2. Atrial Arrhythmia

Just like ventricular arrhythmias, atrial fibrillation is under the influence of ANS. The increased sympathetic tone has been noted to enhance the risk of atrial fibrillation [63]. Many trials use low-level vagus nerve stimulation (LLVNS) due to its significant results in comparison to high-level vagus nerve stimulation (HLVNS) [41,64]. More studies have shown that LLVNS does not induce bradycardia compared to HLVNS. This could be attributed to HLVNS being run at a frequency of >20, thus causing bradycardia (>60%) and accompanying an increase in AVN conduction time [41,64]. Furthermore, low-level tragus stimulation (LLTS) provides a form of LLVNS. A transcutaneous form of VNS is LLTS (presented in Figure 4), which is non-invasive in nature [65] and stimulates the auricular branch of the vagal nerve. Wang Z et al. (2014) demonstrated that LLTS attenuates left ventricular remodeling in dogs recovering from MI [33]. Vagal stimulation in canines via LLVNS application was shown to regulate AF [66]. The Alpha7nACh-mediated cholinergic pathway appears to be involved [66]. 

Recently, a randomized control trial (RCT) conducted by Stavrakis et al. (2020) used LLTS to attenuate paroxysmal AF in humans [36]. They observed an 85% decrease in AF and a 23% reduction of TNF-α in those using LLTS compared to controls [36]. The trial emphasized the VNS’s ability to control heart rate (detailed in Table 2) dynamics in AF patients, similar to those seen in patients with ventricular arrhythmias. Frequency alterations were the primary domain in which changes were seen [36]. LLVNS has also been tested in postoperative patients undergoing cardiac surgery for the prevention of AF [42]. Several studies have used invasive LLVNS, which targeted the ganglionated plexus located on the epicardial surface of the heart, to target cardiac arrhythmias [67]. The reduction in the incidence of postoperative AF ranged from 25% to 7% in the active group [42]. These studies also demonstrated a significant decrease in inflammatory markers, including IL-6, TNF-α, vascular endothelial growth factor (VEGF), and epidermal growth factor (EGF) [42]. Another RCT following a similar invasive LLVNS technique, involving the innervation of the preganglionic vagal nerve along the lateral region of the superior vena cava, demonstrated a decrease of 24% in the incidence of postoperative AF. This could partially be attributed by the decreased inflammatory response [40]. More details on the studies are provided in Table 2.

#### 3.1.3. Ventricular Arrhythmias

Ventricular arrhythmias have been correlated to cause a significant reduction of vagal tone (parasympathetic), a mechanism that allows for management via VNS [68]. A recent study model was created on rabbits with induced long QT-associated arrhythmias, which showed that VNS attenuated the proarrhythmic condition created by increased sympathetic tone [69]. VNS application in a rat model demonstrated its ability to act on heart rate (detailed in Table 2) and rhythm via the aid of G protein–gated inwardly rectifying K+ (GIRK) channels [38]. Furthermore, GIRK removal in the trial displayed no significant response of VNS in their rats, thus indicating the need for GIRK as a tool for VNS action [38]. The significance of the GIRK channel is also seen in another study where it was demonstrated to be a primary controller for heart rate dynamics via VNS [39]. Another study which focused on the effects of ANS in initiating ventricular arrhythmias was performed on innervated rabbit hearts. Results of this study demonstrated a reduction in the current required to induce ventricular arrhythmia via sympathetic nerve stimulation, while VNS achieved the exact opposite [68]. This entails a direct anti-arrhythmic effect, as the anti-fibrillatory effects occurred without any background sympathetic activity. 

Optical mapping was used to show a reversal of the ventricular repolarization direction via nerve stimulation. Anti-cholinergic pharmacological treatment results in different directional patterns of current flow [68]. A study on pigs and sheep found that VNS directly regulated left ventricular function [37]. It was observed that VNS affected ventricular myocytes and the accompanying increased electrical heterogenicity was associated with a significant decrease in blood pressure, thus potentially inducing ischemia in organs. Additionally, VNS was shown to positively impact the animal’s heart following cardiac arrest [37]. The presented positive impact involved the expansion of the action potential duration (APD) and the effective refractory period (ERP), allowing for reduced intracellular calcium, which resulted in a reduction of ventricular motion through reduced contractility [37]. VNS’s ability to control APD and ERP enables it to alter heart rate dynamics in addition to having GIRK channels as a primary controller. This allows for the possibility of VNS usage to enhance cardiac function both as a treatment and as a physiologically enhancing tool. More details on the studies are presented in Table 2.

#### 3.1.4. Cardiac Arrest

An experiment conducted on rats by Choudhary et al. demonstrated that threshold-adjusted VNS (tVNS) animals had a significantly improved survivability rate of up to 72 h after cardiac arrest in comparison to control rats. tVNS had also shown improvement in post-heart injury as the injured rats had lower troponin one levels compared to the control rats [43]. In rats receiving CPR for cardiac arrest, the application of VNS increased the success rate for return of spontaneous circulation (ROSC) by up to 9%. Furthermore, an improved 72-h survival rate was identified in the VNS group. The anti-arrhythmogenic effects of the VNS—acting via the α7nACh receptors—reduced duration of CPR. VNS appears to attenuate the rate of arrhythmias independent of its action on the heart rate (detailed in Table 3) [45]. Results also demonstrated a reduction in the number of electric shocks required to achieve ROSC in CPR, possibly due to its inhibitory effect on the sympathetic autonomic nervous system (ANS), which reduces the number of failed attempts in defibrillation [45]. 

A model involving the asphyxial cardiac arrest of rats similar to that of post-cardiac arrest syndrome (PCAS) has been established [44]. PCAS is a syndrome which includes myocardial dysfunction, microcirculatory dysfunction, global brain injury, increased vulnerability to infection, and persistent advancing pathology [44]. Vagal stimulation in the model attenuates systemic and local inflammatory responses to myocardial ischemia reperfusion injury [44]. These investigators demonstrated that mitochondrial dysfunction was ameliorated after ROSC with the aid of VNS at both the 48-h and 72-h mark of PCAS. In addition, after 6 h from ROSC, the components of the mitochondria, including leak respiration and oxidative phosphorylation capacity of complex I (OXPHOS CI) and electron transport system (ETS) were reduced. These results suggest that VNS may be a good therapeutic tool that has the potential to resuscitate patients in cardiac arrest and heart attacks. More details on the studies are presented in Table 3.

#### 3.1.5. Heart Failure

Heart failure has been recognized to have major implications with the ANS of their individuals, displaying activation of the sympathetic nervous system which causes an imbalance in the vagal tone, autonomic dysfunction and consequently worsens the disease [70]. Hyperactivity of the sympathetic nerves has been shown to advance the dysfunction of left ventricular diastole and increase cardiovascular risk through a lack of homeostatic control through the ANS [70]. Recent evidence in basic and clinical studies points at changes in chemical mediators and central sites that result in altered regulation of sympathetic outflow, thus acting as causes for sympathetic activation [70]. A preclinical study model was created on canines, where they identified the autonomic role of heart failure, and their model involved high-rate ventricular pacing for 8 weeks that induced heart failure whilst maintaining it during that time span, with concomitant use of chronic VNS during the trial [51]. The results of VNS application when compared to the controls led to improvements in cardiac autonomic control whilst attenuating heart failure development [51]. Moreover, improvements were seen in canines that received VNS. These include reductions in left ventricular end-systolic and end-diastolic volume, and improvements in left ventricular ejection fraction (EF), baroreflex sensitivity and heart rate variability [51]. In addition to the previously mentioned anti-inflammatory actions of VNS, further reductions in plasma norepinephrine, angiotensin II and C-reactive protein levels were observed [51]. Another study on canines used VNS and beta-blockade as a mean of therapy for HF compared to stand alone beta-blockade therapy for a span of three months. An improved left ventricular systolic function with an increased EF was seen in canines that received VNS [52]. Overall, not only do these studies suggest that VNS aids left ventricular function in heart failure, but also in VF [37], thus underlining the broad cardio-protection provided by VNS.

A large trial that is known as INOVATE-HF (Increase of Vagal Tone in Heart Failure), was done with 707 patients which assessed the efficacy and safety of VNS in patients with HFrEF [6]. Despite results showing no change in the rate of death or heart failure events in chronic patients, VNS improved quality of life (QOL), 6-minute walk distance and New York Heart Association (NYHA) functional class. Both QOL and NYHA functional class showed improvements in a RCT of NECTAR-HF (NEural Cardiac TherApy foR Heart Failure), which involved 96 patients despite having no effect on cardiac remodeling or function in symptomatic heart failure patients [50]. Another trial known as ANTHEM-HF involved 59 HFrEF patients for over 10 weeks and showed that VNS can modulate the heart in both left- and right-sided stimulation and is widely tolerated by the individuals [49]. These results lay a strong foundation for the usage of VNS in clinical practice.

LLTS, previously noted for its attenuating effect of remodeling on canines with atrial fibrillation [33], had also presented with the same effects on a rat model of HFpEF [47]. The same rat model had also demonstrated an improvement in diastolic function with a decrease in inflammatory markers in comparison to the shams group [47]. Furthermore, the capabilities of LLTS were significant in a RCT involving HFpEF patients, as it improved left ventricular global longitudinal stain (LV GLS), a marker of early detection of left ventricular systolic dysfunction [48]. A recent study was conducted by Stavrakis et al. (2022) on HFpEF patients with at least 2 or more comorbidities. The subjects were randomized into either a sham group (earlobe) or an active group (tragus) for tLLVN (Frequency: 20 Hz, 1 mA below discomfort level), which was conducted daily for 1 h at a time span of 3 months [46]. Results showed significant improvements in both inflammatory function and GLS, as well as a correlation of TNF-α reduction with an improvement in GLS and a significant increase in the QOL of the active group [46]. More details on the studies are presented in Table 4.

### 3.2. sVNS Methods

To further enhance the desired effects of VNS, a recent study assessed the effects of chronic ivabradine therapy (a specific *I*_f_ blocker) on cardiac neuromodulation in healthy rats [71]. They experimented with 32 rats, where 20 were pre-medicated with ivabradine (10 received vagus nerve stimulation and 10 didn’t), and 12 were not pre-medicated with ivabradine (6 received vagus nerve stimulation and 6 didn’t). The results showed how chronic ivabradine therapy diminished the hemodynamic response of vagus nerve stimulation by showing no significant change in the heart rate of pre-medicated rats with ivabradine compared to the non-pre-medicated group. Additionally, chronic ivabradine administration increased vagal tone and shifted the sympatho-vagal balance towards vagal dominance. Theoretically, chronic use of ivabradine would allow for continued anti-inflammatory effects via the VNS, without significant changes in heart rate variability [71].

Having discussed the different applications of vagal stimulation, presented here are the different modalities that have been used for VNS application (Table 5). These include:

#### 3.2.1. Fiber-Selective Stimulation

Fiber-selective stimulation is of great interest for its cardiovascular applications to increase selectivity and reduce side effects [10]. VNS application for cardiac neuromodulation requires activation of small fibers (types B and C), whereas VNS application for epilepsy requires activation of larger fibers (type A and B) [10]. Since smaller fibers have a higher threshold for activation, they require a higher amplitude current to activate [72]. Therefore, it can be predicted that selective cardiac stimulation would have more side effects in comparison to when it is used for epilepsy.

Currently, three different techniques are being studied for selective fiber stimulation of the vagus. These are anodal block, depolarizing pre-pulses, and slowly rising pulses [25]. The anodal block technique ensures directional fiber activation in nerve stimulation [73]. In contrast, the conventional VNS stimulation method stimulates larger nerve fibers (A and B) first and then the smaller ones (B and C) [74]. Anodal block stimulation is used to overcome this obstacle, which hyperpolarizes the anode, thus blocking action potential propagation along the type A fibers [75]. A study conducted by Tosato et al. (2007) explored anodal block in the selective control of heart rate of porcine models [58]. They demonstrated a successful reduction in heart rate while reducing laryngeal side effects up to 77% [58]. A different study conducted by Vuckovic et al. (2008) compared the three techniques for activating vagal nerve fibers in pigs [25]. They showed that the anodal block requires the highest currents, while the depolarizing pre-pulses technique had its charge per phase within the safe limits [25]. The authors of the study endorsed depolarizing pre-pulses as their preferred method due to this reason. Furthermore, they revealed that despite the depolarizing pre-pulses technique being the optimal choice regarding charge per phase and maximum required current, it is the most sensitive to changes in the current amplitude out of all three methods [25].

Another experiment demonstrated hemodynamic improvement after selective atrioventricular node (AVN) vagal stimulation using biphasic rectangular stimuli in adult mongrel dogs with atrial fibrillation (AF) [59]. Moreover, a study created on rats with hypertension and prone to stroke involved a fiber-selective technique of VNS [55]. The results revealed left-sided cervical VNS as the stronger side for inducing bradycardia, tachypnea, and hypotension compared to right-sided cervical stimulation. Likewise, the rectangular impulses only affected heart rate responses but not the respiratory ones and not the mean blood pressure of the rats; no form of anodal blocking was seen [55]. Despite the literature advocating for this stimulation method, more trials are needed to form a standardized application method.

#### 3.2.2. Spatially-Selective Stimulation

Despite fiber-selective stimulation proving to be more popular [10], spatially-selective stimulation is the preferred technique when activating larger nerve fibers that are difficult to avoid [23]. Generally speaking, separating these two techniques (fiber-selective and spatially-selective) is only partially possible, as protocols that require spatial-selectivity may require some degree of fiber-selectivity and vice versa [25]. A study conducted by Ordelman et al. (2013) was the first study exploring the spatially-selective VNS technique. They used a multi-contact cuff that achieved 50% more efficacy in cardiac neuromodulation than conventional techniques [57]. Blanz et al. (2022) recently explored the effect of the spatial selectivity of VNS on changes in heart rate and the unwanted side effects in pig models [53]. The study reported a relationship between the functional anatomy of the vagus nerve and the functional effects of spatially-specific vagal nerve stimulation [53]. There are location-specific differences in saturation and thresholds for the responses of the sVNS in both in vivo and silico studies [53]. There are limitations to this technique, as sVNS can activate nerve fibers that are spatially away from the fibers being stimulated by spatially-specific VNS [10]. In summary, spatially-selective and fiber-selective techniques of VNS usually go hand in hand, and this improves their individual limitations [10].

#### 3.2.3. Kilohertz Electrical Stimulation Block (KES)

The kilohertz electrical stimulation (KES) block was first demonstrated by Patel and Butera (2015) [56] in the sciatic nerve of a frog [56]. Next, they applied it to the vagus nerve [56]. KES block is a technique in which the propagation of action potentials in nerves is inhibited by electrical stimulations of a value not less than 5 kilohertz (kHz) [10]. The traditional KES block technique aims at the entire nerve, providing directional selectivity like the anodal block technique discussed above. Therefore, the KES block has been shown to achieve fiber-selective stimulation [56]. However, it is unclear what advantages KES has over the anodal block technique in sVNS [10]. A recent review by Neudorfer et al. (2021) demonstrates that the KES technique in the stimulation of the nervous system is associated with challenges and must be considered in experimental paradigms [76]. Further studies on the KES block technique for VNS are required to demonstrate its efficacy and to avoid subsequent complications derived from a complete nerve block.

#### 3.2.4. Neural Titration

Neural titration is another technique for sVNS, first presented by Adrell et al. (2015). It demonstrates the opposite effects of efferent and afferent fibers on the modulation of heart rate in dogs [26]. Stimulation of both afferent and efferent fibers of the vagus simultaneously leads to the bradycardia effect from the efferent fibers canceling out the tachycardia effect from the afferent fibers [26]. This dynamic equilibrium has been termed “the neural fulcrum” [26]. Parameters of the stimulation varied for at least 14 months, but an average of 30 days are required to find optimal parameters for each dog [26]. Therefore, carrying out a clinical study using this tool is difficult, especially as Adrell and colleagues used a very controlled environment for an extensive period.

#### 3.2.5. Transcutaneous—Vagus Nerve Stimulation (tVNS)

The tragus and the cymba concha region of the ear acts as a waypoint to cardiac neuromodulation of the heart [27]. Different stimulation patterns will be elicited when different receptors are stimulated. For example, a tingling sensation is seen when Aβ (responsible for the cutaneous mechanoreceptor and touch sensation) receptors are stimulated, whereas painful sensations will be observed with the stimulation of Aδ (responsible for cutaneous pain and temperature sensation) receptors. This has also been documented across different trials [77,78]. A recent study by Yokota et al. (2022) explored different tVNS frequencies and currencies in 35 healthy adults by continuous left cymbal concha stimulation for 1 min [35]. Their results suggested that the higher the frequency, the stronger the afferent vagal nerve signal transmission. A relation between the duration of stimulation and the effectiveness of tVNS was also noticed in reducing the heart rate. Yokota and colleagues suggested that, to reach an effective and optimal dose in clinical application of tVNS, specific stimulus parameters and sex differences should be taken into consideration. This includes a stimulation frequency of 100 Hz, current intensity of 3.0 mA, and over 250 μs to reduce heart rate effectively [35].

A limitation of the LLTS application is that the patients will have concomitant tingling sensations [77,78,79]. This method is generally safe, and no undesirable effects have been noticed with its application except in the study of Stavrakis et al. (2020) [36]. LLTS application induced mild skin lesions, but this was mainly due to the irritation of the clips providing the stimulation [36]. In a RCT study, patients presenting with ST-segment elevation myocardial infarction (STEMI) were treated with PCI. The patients were randomized into a LLTS group and a shams group. LLTS was applied for 2 h after reperfusion. Patients were followed up for seven days continuously. The results of LLTS usage involved reductions in cardiac biomarkers, inflammatory markers, and N-terminal pro–B type natriuretic peptide. Furthermore, improvements in left ventricular ejection fraction (which tentatively indicates a reduced infarction size) were observed [54]. Finally, due to the transcutaneous nature of LLTS application, several systemic side effects, which are observed in other forms of sVNS, are avoided. LLTS appears to be safe and highly effective in clinical conditions.

The results explored the cardioprotective abilities of VNS and the different models for selective stimulation. The evidence supports that VNS has cardioprotective abilities, primarily with arrhythmias and heart failure patients. Despite the collected evidence providing different theories on its mechanism, no set stone mechanism has been established for its mode of action. Furthermore, the novelty of this modality and its uses provides difficulty in understanding it.

## 4. Discussion

### 4.1. Cardiac Diseases

#### 4.1.1. Myocardial Ischemia

Acute myocardial infarction (AMI) is one of the prominent causes of death worldwide. It arises due to the occlusion of the coronary arteries (one or more) either through coronary heart disease or coronary artery disease, thus reducing perfusion and leading to irreversible heart muscle damage. It can lead to other worsening conditions like arrhythmias, cardiac arrest, and many more. AMI is responsible for over 15% of yearly deaths worldwide, with its prevalence being more amongst men than women [80]. Currently, incidence rates are surging in developing countries in South Asia, parts of Latin America and Eastern Europe. Recent studies have found that modifiable risks accounted for 94% of women and 90% of men as a cause for AMI; these risks include triglycerides levels, cholesterol, LDL (Low-density lipoprotein), obesity, smoking, and hypertension. Approximately 70% of the fatal cases were caused by atherosclerotic plaques [81,82]. AMI is a time-sensitive case in which minutes saved by fast treatment can drastically increase the survival rate of damaged tissue. Current treatments include non-steroidal anti-inflammatory drugs (NSAID), antiplatelets, beta-blockers, angiotensin-converting enzyme inhibitor (ACE-i), and percutaneous coronary intervention (PCI) [83]. With these treatments, the reperfusion of the damaged tissue could lead to 3 significant implications that cause ischemia/reperfusion (I/R) injury [84]. Firstly, restoring physiologic pH and Ca^2+^ overload could induce changes to mitochondrial membrane potential and ATP depletion. Secondly, AMI causes the opening of mitochondrial permeability transition pores (mPTP) that consequently induce the generation of reactive oxygen species (ROS). ROS causes DNA damage that can induce apoptosis and stimulates pro-inflammatory secretions that enhance the I/R injury [84]. Finally, neutrophil accumulation is attributed to chemo-attractants at the injury site [85], which are all enhanced by reperfusion, mainly through PCI. VNS usage against these mechanisms has shown potential effects for treatment (Table 1).

#### 4.1.2. Atrial Arrhythmias

Atrial arrhythmia, the most common form being atrial fibrillation (AF), is the leading cardiac cause of stroke globally [86]. The prevalence of atrial fibrillation surges with age and is increasing noticeably worldwide, despite it affecting 1% of the global population. It affects 9% of those 75 years or older, which will increase as the worldwide population survives to older ages [86]. Atrial fibrillation arises through cardiac modulation of atrial walls, resulting in a deranged rhythm. The etiology is largely from underlying cardiovascular disease and other conditions triggering atrial fibrillation. Triggers include atrial ischemia, inflammation, alcohol or illicit drugs, hemodynamic stress, advanced age, genetic factors, and neurological or endocrine disorders [87]. Atrial fibrillation management depends on its etiology, hemodynamic stability, and risk stratification. In cases involving hemodynamically unstable patients, they require carrying out immediate cardioversion with anticoagulants.

#### 4.1.3. Ventricular Arrhythmias

Ventricular arrhythmia, also known as ventricular tachycardia, is the presence of irregular heart rhythms in the ventricles that causes tachycardia when present. It is one of the major causes of sudden cardiac death [88]. The most common form of ventricular arrhythmia is ventricular fibrillation (VF), causing 50% of cardiac deaths, with an estimated death rate of 300,000 in the United States [89]. VF arises via injury currents that results in infarcted tissue created by AMI. Consequently, it leads to the development of increased extracellular potassium causing partial depolarization of the myocardium [89]. Injury currents mixed with healthy currents trigger spontaneous activity that presents as arrhythmias [89]. Other causes of injury currents include acquired and inherited channelopathies, digitalis toxicity, infiltrative cardiomyopathy, electrolyte imbalances, and skeletal heart diseases [90]. Management for VF involves the use of medications and implantable cardiac defibrillators, with worsening cases requiring the use of catheter ablation.

Both atrial and ventricular arrhythmia types require more trials to better understand the neurological pathways involved. More trial are required to increase our understanding of the pathophysiology as well as for dealing with limitations provided by the variable anatomical distribution of the vagus nerve across persons, with the end goal of developing better and safer outcomes for future use.

#### 4.1.4. Cardiac Arrest

Cardiac arrest is the sudden stop of the person’s heart with no signs of circulation or normal breathing. Most cardiac arrests are due to underlying structural diseases, 70% of which are caused by ischemic coronary disease, and the remaining include congestive heart failure, arrhythmogenic right ventricular dysplasia, as well as nonstructural conditions like Wolf-Parkinson-White syndrome (causes the heart to beat abnormally fast for a duration of time) and Brugada syndrome [91]. 70% of all sudden cardiac death in the United States is due to underlying coronary heart disease [92]. An approximation for the global annual incidence of sudden cardiac death would be in the array of 4–5 million cases per year, with it being 180,000 to 250,000 per year in the United States [88]. Treatment for cardiac arrest involves multiple methods, including cardiopulmonary resuscitation (CPR) with chest compressions, defibrillation, ventilation, medications, other advanced resuscitation methods, and long-term management. It involves three stages, the first phase requires defibrillation, which is very effective as it is during the electrical stage for the first 5 min, followed by CPR between 5 to 10 min from the arrest at the circulatory stage, and eventually, the most lethal phase which is the metabolic phase that starts after 10 min from the event with a high mortality rate [93]. All of these showcase the time-sensitive nature of cardiac arrests.

#### 4.1.5. Heart Failure

Heart failure (HF) is a clinical syndrome in which the heart is incapable of pumping sufficient blood around the body. Heart failure derives from the insufficient ventricular filling or ejection of blood from the heart chambers, due to defects in the structure or function of the myocardium [94]. Incidence of heart failure in the United States and Europe was deemed to be in the range of 1 to 9 per 1000 person-year, with evidence of a reduction in prevalence from the 1990′s [95]. The majority of heart failure patients exhibit with many comorbidities and the rate of three or more comorbidities presenting in patients has increased from 68% to 87% (2002 to 2014, respectively) [96]. Typically, heart failure is classified as either chronic or acute, but clinically it is referred to as: (1) heart failure with mildly reduced ejection fraction (HFmrEF), (2) heart failure with reduced ejection fraction (HFrEF), (3) heart failure with improved ejection fraction (HFimpEF), and (4) heart failure with preserved ejection fraction (HFpEF) [94]. The dominant cause of heart failure is reduced left ventricular myocardium function, with other causes involving the great vessels, heart valves, pericardium, myocardium, and endocardium of the heart [94]. The syndrome involves pathogenesis via hemodynamic overload, ventricular remodeling, ischemia related dysfunction, excessive neuro-hormonal stimulation, abnormal myocyte calcium cycling, accelerated apoptosis and many more [97]. Treatment of heart failure depends on the etiology for each patient, as it involves both non-pharmacological and pharmacological treatment, in addition to surgical repair and implantation of cardiac devices [98].

### 4.2. Cellular Mechanism of VNS

Findings seen in Yokota et al.’s (2022) use of tVNS showcases a shift of the vagus towards parasympathetic balance [35]. The theory is that, in addition to the stimulation role played by VNS, it acts a neuromodulation technique for the vagal afferent projections. VNS has a role in the peripheral autonomic system through the nucleus tractus solitarius (NTS). The NTS acts as the first synaptic point for autonomic afferent projections in the central nervous system. In turn, the NTS recruitment could activate the excitatory input to the caudal ventrolateral medulla which inhibits the rostral ventrolateral medulla, the source for excitation to the sympathetic efferent [99].

Poppa et al. (2022) discovered that VNS neuromodulation in humans involves the insula and functionally related regions. Through electroencephalogram (EEG) and magnetoencephalography (MEG) scans, the related functional regions were identified as such: the somatosensory, cingulate, ventromedial prefrontal and orbitofrontal cortices. Moreover, lateralization of response in the NTS may reflect its organization of the ascending pathways [100]. The study used heart-evoked potentials (HEP) in ECG as a measure that reflects neural processing of cardiac dynamics through multiple nerves, primarily the vagal, and noted that it involves the insula and operculum of the brain. The HEPs is believed to be a direct reflection of transduction to the baroreceptors, intrinsic cardiac neurons, and cutaneous mechanoreceptors [100]. During the experiment, HEP involvement was elicited using VNS. In theory, this provides strong correlation to VNS’ ability to target the heart whilst modulating high order autonomic control to the favored effects. Nonetheless, more research is needed as not much detail on its physiology is present in current literature.

### 4.3. Limitations and Bias

Certain limitations should be considered in this review. These include gathering only English language studies and studies with full-text access. Studies with high relevance and importance could have been missed due to this reason. Selection bias should also be acknowledged. Lastly, as the VNS is a newly emerging tool in cardioprotective medicine, limited studies are in the literature regarding its usage and application.

## 5. Conclusions

The review identified the effects of VNS on cardiac pathologies. A strong relationship was evident between VNS and its ability to modulate inflammation and heart dynamics. Understanding the mechanisms by which VNS alters the physiology and pathophysiology of cardiac diseases is key for the future use of VNS towards cardio-protection. More studies should focus on LLTS, as literature proves its potential for clinical use. Other forms of sVNS prove to be difficult to proceed upon, thus requiring prolonged and more in-depth experiments to further enhance its stance towards cardio-protection.

## Figures and Tables

**Table 1:** Studies on VNS usage for myocardial infarction (N/A—Not Available, I/R—Ischemia Reperfusion, VNS—Vagus Nerve Stimulation, MI—Myocardial Infarction, LV—left ventricle, TNF α—Tumor Necrosis Factor Alpha).

Study	Year	Model	CVS Disease	Characteristics	Device	VNS Tool	Outcome/Result
Yi et al. [29]	2016	Rat	I/R injury	I/R rats subjected to occlusion of the left anterior descending artery for 30 min, followed by reperfusion for 4 h	Grass technologies	Rectangular electrical pulses (10 Hz for 2 ms)	Significant reduction in infarct size following I/R injury.
Beaumont et al. [30]	2015	Pig	MI	Surgical induction of MI by ligation of ventral descending coronary artery and associated vein	Cyberonics	Bipolar VNS electrode (cyclical stimulation 14 s on and 48 s off for 80 days, frequency 20 Hz, 500 μs pulse duration)	Focal mitigation of remodelling of the intrinsic cardiac nervous system
Zhang et al. [31]	2014	Dog	I/R injury	Surgical occlusion of the left anterior descending artery for 1 h, followed by reperfusion for 6 h	SEN-7103, Nihon-Kohden	Special silver-argentic chloride stimulation electrode (frequency 10 Hz, pulse width 0.5 ms and stimulation strength 1.5–3 V)	Inhibition of pro-inflammatory products like TNF-α
Shinlapawittayatorn et al. [32]	2014	Pig	I/R injury	Occlusion of the left anterior descending artery for 60 min, followed by reperfusion for 120 min, with VNS received either after 30	N/A	Bipolar pacing lead and anchor lead for cyclical stimulation 20 s on and 30 s off (frequency 20 Hz, current 3.5 mA)	Cardioprotective effects during ischemia but not reperfusion.
Wang et al. [33]	2014	Dog	MI	Harris 2-stage occlusion of the left anterior descending artery (partial occlusion for 20 min then completely for 3 h)	N/A	Custom-made stimulator for cyclical low-level transcutaneous stimulation on bilateral tragus, 5 s on and 5 s off (frequency 20 Hz, and pulse width 1 ms)	Significant improvement in alleviated cardiac fibrosis, cardiac function, and attenuated LV remodeling
Kong et al. [34]	2011	Rat	MI	Occlusion of the left anterior descending artery for 240 min continuously	N/A	Bipolar electrodes attached to the cardiac end of right vagus nerve (frequency 5 Hz, voltage 2–6 V)	Significant attenuation of TNF-α signaling pathway

**Table 2:** Studies on VNS usage for atrial fibrillation (AF), ventricular fibrillation (VF), and heart rate modulation. (GIRK—G protein–gated inwardly rectifying K+, LLTS—Low Level Tragus Stimulation, N/A—Not Available, TNF α—Tumor Necrosis Factor Alpha, VNS—Vagus Nerve Stimulation).

Study	Year	Model	CVS Disease/Parameter	Characteristics	Device	VNS Tool	Outcome/Results
Yokota et al. [35]	2022	Human	Heart rate modulation	Healthy adults monitored with two different ECG devices	NEMOS	hemispheric titanium-stimulating electrodes on cymba concha for transcutaneous stimulation (experimented with different sets of stimulation frequencies at 100 Hz, 25 Hz, 10 Hz, 1 Hz, and 0 Hz)	A more significant decrease in heart rate at 100 Hz signifies a frequency-dependent manner for the vagal transmission
Stavrakis et al. [36]	2020	Human	Atrial fibrillation	Paroxysmal AF patients, documented by an ECG with in a 3-month span (2 separate occasions)	Parasym device	Ear clip onto the tragus for low level transcutaneous stimulation (frequency 20 Hz and pulse width 200 ms) for 1 h continuously every day for 6 months	85% decrease in AF and a 23% reduction of TNF-α in those using LLTS compared to controls
Naggar et al. [37]	2018	Pig and sheep	Ventricular fibrillation	VF was induced by large, low impedance electrodes on the ventricles with a direct current of 3–5 volts for several seconds	A-M Systems model 2100	Platinum strip electrodes. Bilateral innervation of cervical vagus nerve (frequency 50 Hz, current 1–10 mA, voltage 3–5 V)	A direct electrical effect on the ventricles
Lee et al. [38]	2018	Rat	Heart rate modulation	Healthy rats. Samples and recordings taken before, during, and after VNS.	Demipulse Model 103 VNS pulse generator	Helical lead bipolar cuff electrodes on the right cervical vagus nerve (frequency 10 Hz, current 0.25 mA, 500 μs pulse duration) for 1 min	GIRK proved feasible as a tool for VNS action
Lee et al. [39]	2018	Rat	Heart rate modulation	Healthy rats. Samples and recordings taken before, during, and after VNS.	Cyberonics	Gaussian distribution of stimulation frequencies for stochastic VNS, on the right cervical vagus nerve (frequency 10,20 or 30 Hz, current 0.1 mA) for 2 min	Further attenuation heart rate with stochastic VNS compared to Standard VNS method
Stavrakis et al. [40]	2017	Human	Atrial fibrillation	Patients undergoing cardiac surgery, including coronary artery bypass graft and valve surgery	N/A	Bipolar wire for low level vagus nerve stimulation, adjacent to the superior vena cava (frequency 20 Hz, pulse duration 0.1 ms) upto 72 h	Decrease of 24% in the incidence of postoperative atrial fibrillation in the active group compared to the sham controls (12% vs. 36%)
Stavrakis et al. [41]	2015	Human	Atrial fibrillation	Paroxysmal AF patients that were referred to an electrophysiology lab for AF ablation	Grass S88 stimulator	A flat metal clip onto the tragus for low level transcutaneous stimulation (frequency 20 Hz, square wave 1 ms)	Suppression of atrial fibrillation and decreased inflammatory cytokines
Rossi et al. [42]	2012	Human	Atrial fibrillation	Patients undergoing coronary artery bypass graft	LabView system (Streamline Convenience model 6495 Medtronic)	Bipolar temporary wire for low level vagus nerve stimulation (frequency 50 Hz, pulse duration 1 ms)	Reduced incidence of postoperative atrial fibrillation from 25% to 7% in controls, with significant decrease in inflammatory cytokines

**Table 3:** Studies on VNS usage for cardiac arrest (CA). (N/A—Not Available, VNS—Vagus Nerve Stimulation, CPR—Cardio Pulmonary Resuscitation).

Study	Year	Model	CVS Disease	Characteristics	Device	VNS Tool	Outcome/Results
Choudhary et al. [43]	2022	Rat	Cardiac arrest	12 min of untreated asphyxia-CA via vecuronium bromide injection	N/A	Threshold adjusted VNS, biphasic rectangular electrical pulses left cervical vagus nerve	Significant improvement in survivability rate to 72 h
Kim et al. [44]	2022	Rat	Cardiac arrest	450 s of untreated asphyxia-CA via vecuronium bromide injection	Model 2100 isolated pulse stimulator, A-M Systems	Platinum electrode for stable electrical stimulation, left cervical vagus nerve (frequency 1 Hz, current 1 mA, pulse duration 10 ms) for 3 h	Relieve of mitochondrial dysfunction after post cardiac arrest syndrome
Sun et al. [45]	2018	Rat	Cardiac arrest	Ventricular fibrillation was induced and untreated for 8 min, leading to CA	Model 8002A pulse generator; Hewlett-Packard	Electrical rectangular pulses, right cervical vagus nerve (frequency 10 Hz, pulse duration 0.5 ms, voltage 2–6 V)	Improved CPR outcomes

**Table 4:** Studies on VNS usage for heart failure (HF). (EF—Ejection Fraction, N/A—Not Available, NECTAR-HF—Neural Cardiac Therapy for Heart Failure, NYHA—New York Heart Association, VF—Ventricular Fibrillation, VNS—Vagus Nerve Stimulation).

Study	Year	Model	CVS Disease	Characteristics	Device	VNS Tool	Outcome/Results
Stavrakis et al. [46]	2022	Human	Heart failure	Heart failure patients with preserved ejection fraction and at least 2 co-morbidities	Parasym device	low-level transcutaneous vagus nerve stimulation, Active (tragus) or sham (earlobe), (frequency 20 Hz, current 22.9 ± 13.4 mA, pulse dura-tion 0.2 ms)	Significant improvement in inflammatory cytokines, global longitudinal strain, and quality of life
Zhou et al. [47]	2019	Rat	Heart failure	Dahl salt sensitive rats were feed a high salt diet to induce HF	InTENSity™ Twin Stim^®^	Oppositely charged magnetic electrodes for low-level transcutaneous stimulation (frequency 20 Hz, current 2.0 mA, pulse duration 0.2 ms) 30 min daily for 4 weeks.	Significant improvement in cardiac diastolic dysfunction and attenuation of cardiac inflammation and fibrosis
Tran et al. [48]	2018	Human	Heart failure	Patients diagnosed with diastolic dysfunction via echocardiogram, within 24 months from study enrollment	Parasym device, Parasym Health	Ear clip electrode for low level transcutaneous stimulation (frequency 20 Hz, current 1 mA below the discomfort threshold, pulse width 200 μs)	Improved left ventricular global longitudinal stain (a marker for early recognition of left ventricular systolic dysfunction)
Dali et al. [24]	2018	Sheep	Heart failure	Sheep infarcted to create significant HF	Custom-made	12-pole cylindrical cuff electrode for spatially selective control (frequency 25.6 Hz, current 1.33 mA)	62% reduction in side effects compared to non-selective VNS
Gold et al. [6]	2016	Human	Heart failure	Patients with NYHA class III symptoms of HF	BioControl CardioFit system	Nerve stimulation cuff on the right vagus nerve (current 3.5–5.5 mA)	No reduction in rate of death in HF, but improved quality of life.
Nearing et al. [49]	2016	Human	Heart failure	Patients with NYHA class II or III symptoms of HF	Demipulse Model 103 pulse generator, Cyberonics	Lead placement randomized 1:1 to either the right or left cervical vagus nerve, with cyclical stimulation 14 s on and 66 s off. Initially (frequency 10 Hz, pulse width 130 μs, current 1.5–3.0 mA) then intensity was increased in 0.25 mA steps until it was intolerable	Ability to modulate the heart in both left- and right- sided stimulation
Zannad et al. [50]	2015	Human	Heart failure	Patients with NYHA class II or III symptoms of HF	NECTAR-HF system	Self-sizing bipolar helical lead by cyclical stimulation of 10 s on and 50 s off (frequency 20 Hz, current 4 mA, pulse duration of 300 μs)	Having no effect on cardiac remodeling or function
Zhang et al. [51]	2009	Dog	Heart failure	Dogs underwent 8 weeks of VF (4 weeks to develop HF and 4 weeks to maintain HF)	Cyberonics	Right cervical vagus nerve electrode with cyclical stimulation of 14 s on and 12 s off (frequency 20 Hz, current 0.75 to 2.5 mA, pulse width 0.5 ms)	Improved cardiac autonomic control whilst attenuating HF development
Sabbah et al. [52]	2007	Dog	Heart failure	Dogs with chronic HF undergoing 3 months therapy by the experiment	CardioFit VNES system	Vagus nerve stimulation combined with beta-blockade therapy	Improved left ventricular systolic function with an increased EF of 9.8 ± 0.6% compared to controls

**Table 5:** Studies on different sVNS techniques in various applications. (PCI—Percutaneous Coronary Intervention, KES—Kilohertz Electrical Stimulation, N/A—Not Available, sVNS—selective Vagus Nerve Stimulation, STEMI—ST Elevation Myocardial Infarction, VNS—Vagus Nerve Stimulation).

Study	Year	Model	Application	Characteristics	Device	VNS Tool	Outcome/Results
Blanz S et al. [53]	2022	Pig	Heart rate modulation	Healthy pigs	ImThera	Spatially-selective control, right cervical vagus nerve	Reduced side effects
Yu et al. [54]	2017	Human	Myocardial infarction	Patients with STEMI, who presented with 12 h of symptoms from onset and underwent PCI	S20, Jinjiang	Low-level tragus stimulation (frequency 20 Hz, pulsed duration 1 ms)	Significant improvement in myocardial injury biomarkers, inflammatory responses, and reperfusion-related ventricular arrhythmias.
Stauss et al. [55]	2017	Rat	Hypertension	Stroke-prone, spontaneously hypertensive rats	Grass instruments	Fiber selective control, bilateral cervical vagus nerve (experimented with various frequencies and pulsed durations)	Stronger cardiorespiratory response in left-sided cervical VNS compared to the right.
Patel et al. [56]	2015	Rat	Neuromodulation	Healthy rats	N/A	KES block technique (pulse width 0.2 ms, voltage 5 V)	Possible application of KES block for selective stimulation of nerves
Adrell et al. [26]	2015	Dog	Heart rate modulation	Healthy dogs	PerenniaFlex model, Cyberonics	Bipolar stimulating helical cuff electrodes for neural titration method (frequency 10 Hz, pulse width 500 μs) a range of different frequencies were experimented.	Simultaneous stimulation of efferent and afferent fibers resulted in bradycardia
Ordelman et al. [57]	2013	Pig	Heart rate modulation	Healthy pigs	custom-made	Biphasic pulses for spatially-selective control (frequency 10–50 Hz, pulse amplitude 1–10 mA, pulse width 300 μs) for 60 s	Successful reduction in heart rate and respiratory rate
Vuckovic et al. [25]	2008	Pig	Neuromodulation	Healthy pigs	N/A	Fiber selective control with anodal block, depolarizing pre-pulses, and slowly rising pulses techniques	Anodal block requiring high currents. Depolarizing pre-pulses has safe limits in its charge per phase but most sensitive to current amplitude.
Tosato et al. [58]	2007	Pig	Heart rate modulation	Healthy pigs	N/A	Fiber selective control with anodal block technique	Successful reduction in heart rate with reduced laryngeal side effects by 77%
Wallick et al. [59]	2001	Dog	Atrial fibrillation	Healthy dogs subjected to AF initiated by brief burst of right atrial stimulation then maintained by sinus node fat-pad stimulation for at least 15 min	Master-8, AMPI	Fiber selective control (frequency 20 Hz, current >3 mA, pulse duration 50 μs)	Hemodynamic improvement

## Data Availability

The full-text article includes the dataset used in this systemic review.
